# An epigenetic aging clock for dogs and wolves

**DOI:** 10.18632/aging.101211

**Published:** 2017-03-26

**Authors:** Michael J. Thompson, Bridgett vonHoldt, Steve Horvath, Matteo Pellegrini

**Affiliations:** ^1^ Molecular, Cell and Developmental Biology, University of California, Los Angeles, Los Angeles, CA 90095, USA; ^2^ Ecology and Evolutionary Biology, Princeton University, Princeton, NJ 08544, USA; ^3^ Department of Human Genetics and Biomathematics, David Geffen School of Medicine at UCLA, Los Angeles, CA 90095, USA

**Keywords:** epigenetic clock, biological age, canine, dog, wolf, DNA methylation

## Abstract

Several articles describe highly accurate age estimation methods based on human DNA-methylation data. It is not yet known whether similar epigenetic aging clocks can be developed based on blood methylation data from canids. Using Reduced Representation Bisulfite Sequencing, we assessed blood DNA-methylation data from 46 domesticated dogs (*Canis familiaris*) and 62 wild gray wolves (*C. lupus*). By regressing chronological dog age on the resulting CpGs, we defined highly accurate multivariate age estimators for dogs (based on 41 CpGs), wolves (67 CpGs), and both combined (115 CpGs). Age related DNA methylation changes in canids implicate similar gene ontology categories as those observed in humans suggesting an evolutionarily conserved mechanism underlying age-related DNA methylation in mammals.

## INTRODUCTION

Technological breakthroughs surrounding genomic platforms have led to major insights about age related DNA methylation changes in humans [1-9]. In mammals, DNA methylation represents a form of genome modification that regulates gene expression by serving as a maintainable mark whose absence marks promoters and enhancers. During development, germline DNA methylation is erased but is established anew at the time of implantation [10]. Abnormal methylation changes that occur because of aging contribute to the functional decline of adult stem cells [11-13]. Even small changes of the epigenetic landscape can lead to robustly altered expression patterns, either directly by loss of regulatory control or indirectly, via additive effects, ultimately leading to transcriptional changes of the stem cells [14].

Several studies describe highly accurate age estimation methods based on combining the DNA methylation levels of multiple CpG dinucleotide markers [15-18]. We recently developed a multi-tissue epigenetic age estimation method (known as the epigenetic clock) that combines the DNA methylation levels of 353 epigenetic markers known as CpGs [17]. The weighted average of these 353 epigenetic markers gives rise to an estimate of tissue age (in units of years), which is referred to as "DNA methylation age" or as "epigenetic age". DNA methylation age is highly correlated (r=0.96) with chronological age across the entire lifespan [8, 19, 20]. We and others have shown that the human epigenetic clock relates to biological age (as opposed to simply being a correlate of chronological age), e.g. the DNA methylation age of blood is predictive of all-cause mortality even after adjusting for a variety of known risk factors [21-25]. Epigenetic age acceleration (i.e. the difference between epigenetic and chronological age) is associated with lung cancer [26], cognitive and physical functioning [27], Alzheimer's disease [28], centenarian status [25, 29], Down syndrome [30], HIV infection [31], Huntington's disease [32], obesity [33], menopause [34], osteoarthritis [35], and Parkinson's disease [36]. Moreover, we have demonstrated the human epigenetic clock applies without change to chimpanzees [17] but it no longer applies to other animals due to lack of sequence conservation.

Many research questions and preclinical studies of anti-aging interventions will benefit from analogous epigenetic clocks in animals. To this end we sought to develop an accurate epigenetic clock for dogs and wolves. Dogs are increasingly recognized as a valuable model for aging studies [37, 38]. Dogs are an attractive model in aging research because their lifespan (around 12 years) is intermediate between that of mice (2 years) and humans (80 years), thus serving as a more realistic model for human aging than most rodents. Dogs have already been adopted to model multiple human diseases in gene mapping studies (e.g. squamous cell carcinoma [39], bladder cancer [40]) and cancers are often the cause of age-related mortality in domestic dogs [41].

The maximum lifespan of dogs is known to correlate with the size of their breed [42-44]. Based on previous studies in human [17], we expect that the age acceleration (difference between epigenetic age and chronological age) correlates with longevity. We hypothesize that dogs whose epigenetic age is larger than their chronological age are aging more quickly, while those with negative value are aging more slowly. Thus, we would expect to see a correlation between age acceleration and dog breed size.

We also sought to build an epigenetic clock for gray wolves because alternative age estimation methods have limitations. Gray wolf age estimates have traditionally been conducted through tooth wear patterns, cranial suture fusions, closure of the pulp cavity, and cementum annuli [45, 46]. Based on tooth wear patterns, the age structure of a wolf pack is typically skewed towards younger animals (<1-4 years old), with few individuals >5 years of age [46, 47]. Sexually maturity is reached between 10 months and 2 years of age [48, 49]. In a wild social carnivore, group living often results in high mortality rates. Gray wolves live on average 6-8 years in natural populations, but can live up to 13+ years in captivity with increased reproductive success [45, 46].

## RESULTS

### Data set

We used Reduced Representation Bisulfite Sequencing to generate DNA methylation data of 46 domestic dogs (26 females, 20 males) and 62 gray wolves from Yellowstone National Park (26 females, 36 males). The age distribution of wolves is skewed towards younger animals (Dogs: mean=5 years, median=4, range=0.5-14; Wolves: mean=2.7, median=2, range=0.5-8) due to younger mortality rates in natural populations compared to domestic species, and that estimating the age in wild specimens lacks precision. Additionally, we included 729 humans (388 females, 341 males) with a large age range (mean=47.4, range=14-94).

Based on calculations and criteria described in the Methods section, we constructed a matrix of high confidence methylation levels across 108 canid blood samples. Previous work has shown that there are locus-specific significant methylation differences between dogs and wolves [50]. Here, however, we sought to identify a clock that correlated with age across both canid species; thus, we removed the methylation sites that showed species-specific divergence. This yielded a set of 252,240 CpG sites for our modeling efforts. Of these, 105,521 could be mapped to syntenic CpGs in the human genome (hg19) for functional annotation purposes. Further, a subset of 9,017 sites are measured by the human Illumina 405K array, which allowed us to test for conservation of age correlations between these evolutionarily divergent species (humans, dogs, and wolves).

From these input sets of 10s to 100s of thousands of CpGs, regression models were obtained using an algorithm (see Methods) that selects a much smaller number of CpGs by allowing regression coefficients to go to zero. As the space of possible models is combinatorially vast, there is no guarantee of global optimality of the resulting models, and there are likely a large number of models that would yield comparable results. Thus, we make no assertions of biological significance for the exact identity or number of CpGs in a given model used here.

### Conservation of age-correlated methylation between dogs and wolves

**Figure 1:**
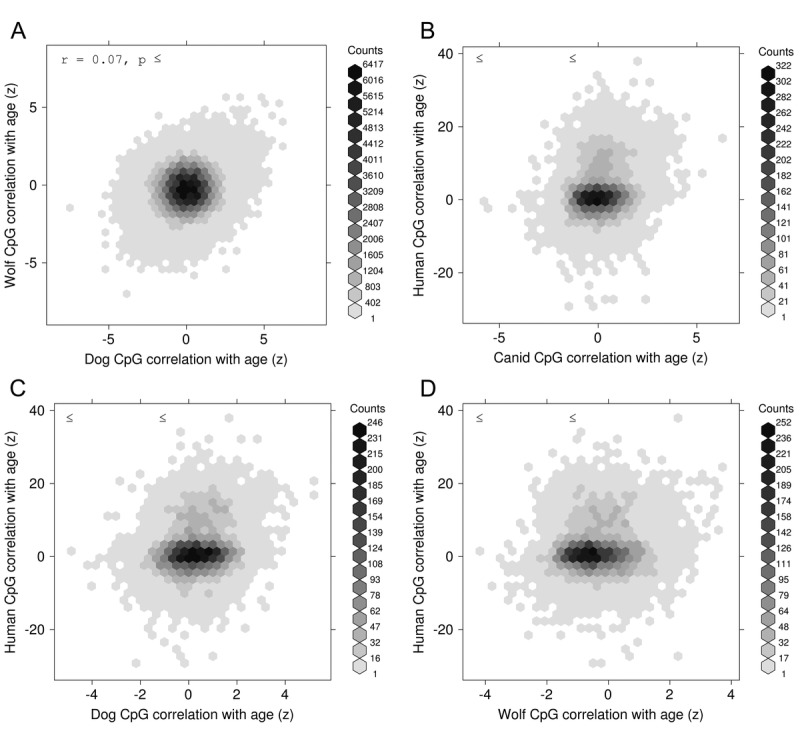
Conservation of epigenetic aging Normalized correlation (z) between age and DNA methylation for CpG sites in one species versus the same correlation computed at syntenic CpG sites in another species. The species comparisons are shown, as follows: (**A**) Wolves versus Dogs, (**B**) Human versus Canid (pooled dogs and wolves), (**C**) Human versus Dogs, and (**D**) Human versus Wolves.

To initially gauge whether it might be possible to create a DNAm age clock for a multi-species group (i.e. canids), we looked at the conservation of age-correlated methylation in the two canid species. The global correlation between the age effects across the two species is small in magnitude (r=0.07, Fig. 1A) which could be due to the following reasons: i) it could reflect poor accuracy of the chronological age estimate in wolves, ii) it could reflect the relatively small sample size, iii) it could reflect that wolves tended to be younger than dogs in our study, i.e. the chronological age distributions differed.

### Conservation of age-correlated methylation between canid species and human

To test for more distant evolutionary conservation of age effects on DNA methylation between canids and humans, we computed age correlations over a set of 729 human blood methylation array samples [6] and examined syntenic locations between the canine (canFam3) and human (hg19) genomes as described in Methods. While the subset of measured DNA-methylation sites common to all 3 species is relatively small (~9000 CpGs), we see that the conservation of age-correlation between “canids” (pooled samples of dogs and wolves) and human is statistically significant, though small in magnitude (r=0.20, p=1×10^-81^, Fig. 1B). This conservation holds for dogs alone (r=0.20, p=6×10^-85^) but is weaker for wolves alone (r=0.11, p=1×10^-25^, Fig. 1C, 1D).

The high correlation between dogs and humans is remarkable because the two data sets were generated on different platforms (RRBS versus the Illumina 450K array).

### Leave one out estimate of the accuracy of the canid epigenetic clock

**Figure 2:**
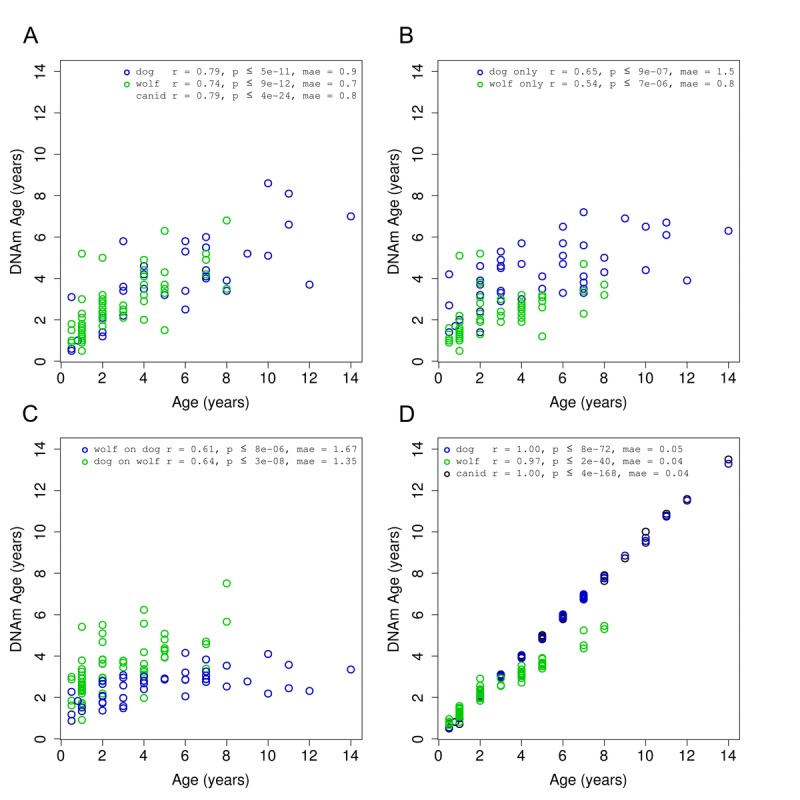
Accuracy of canid age clock DNA methylation age (y-axis) versus chronological age (x-axis) for all canid samples (green = dog, blue = wolf). (**A**) Results obtained using a leave-one-out cross validation over all 108 samples. (**B**) Results obtained in each species separately using a leave-one-out cross validation. (**C**) Results obtained by regressing on all samples in one species and predicting age on samples from the other species. (**D**) Final models for each grouping of samples.

DNAm age (also referred to as epigenetic age) was calculated for each sample by regressing an elastic net on the methylation profiles of all other samples and predicting the age of the sample of interest. In the course of our work, we found that pre-selecting subsets of CpGs was helpful and computationally expedient. This was done by computing correlations between methylation and age and taking only those with absolute correlation above 0.3. These pre-selection steps were also performed in a leave-one-out manner for all cross-validated results presented here. These predictions (in years) were obtained by taking the exponential of the output of the epigenetic aging model where ages were log-transformed prior to regression. We see a strong linear relationship between DNAm age and true age for our 108 canid samples (Fig. 2A). The correlation between predicted and actual ages using leave-one-out cross-validation was 0.8 and the median absolute error was 0.8 (years). The average number of CpGs in the 108 individual regression models was 122.3.

To examine the effects of pooling two species of canids, we performed the same prediction (DNAm age calculation) procedure on dogs and wolves, separately. We find that the performance of these models is lower than the canid model, with dogs showing a correlation of r=0.65 and wolves r=0.54 (Fig. 2B). The average number of CpGs in the dog-only and wolf-only models were 58.5 and 62.9, respectively. These models, on average, contain fewer CpGs than the combined canid models as the smaller number of samples in each subset provides less statistical support for the regression algorithm.

As another means of assessing the robustness of a multi-species clock, we built one clock for each species using all samples in that species and then applied it to all samples in the other species. These clocks have similar correlation to the dog only or wolf only clocks, close to 0.6, utilizing a single regression model with 67 and 41 CpGs for the dog and wolf model, respectively (Fig. 2C).

### Final epigenetic aging clocks based on all animals

To determine the accuracy of our final models, we regressed the penalized elastic net over the set of dogs (41 CpGs), wolves (67 CpGs), and then both combined (115 CpGs) (Fig. 2D). The penalized regression routine (“elastic net”) utilizes an internal cross-validation to select the optimal penalty parameter. While the entire set of canids, and the subset of domesticated dogs could be fit exactly (r=1.0), the wolf data alone was slightly less amenable.

### Age acceleration as a function of dog size

With the largest variation in size among terrestrial vertebrates, the domestic dog not only spends most of its life in an environment and lifestyle like its human companions, but also displays a high similarity of analogues to human disease [51, 52]. Though dog breeds are diverse in nearly every aspect, smaller breeds are known to live longer than larger breeds [42-44]. Recent genomic surveys have identified nine loci linked to canine size determination, with seven of these loci supporting growth, cellular proliferation, and metabolism [53]. Of these, the growth hormone IGF1 has not only been of historic interest as a causative locus controlling body size in mice [54-56], but also has the most significant association with body size [57, 58].

**Figure 3:**
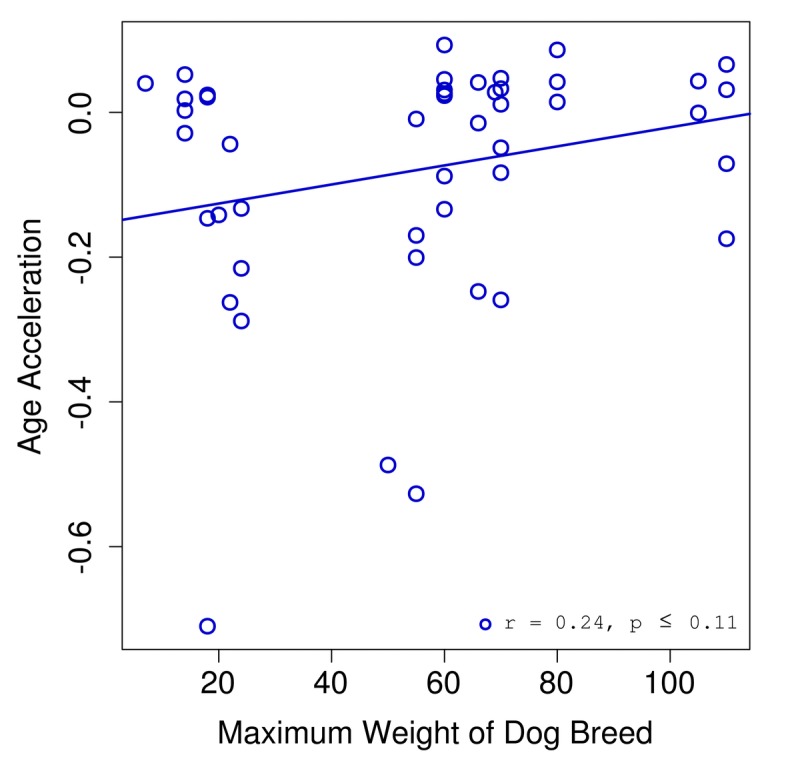
Age acceleration and dog breed Age acceleration (difference between predicted epigenetic age and actual chronological age) is plotted against the maximum weight for the breed of each dog sample.

We found a correlation of 0.25 between age acceleration and breed weight (Fig. 3). Given the limited sample size for dogs (n = 46) we did not reach a significance below the standard threshold of 0.05. However, we expect that a study with a larger cohort might have sufficient power to show that these trends are in fact significant.

### Functional significance of DNAm age sites

As described in Methods, mapping of canid CpGs to the human genome yielded 105,521 sites. We utilized this entire set as “background” and selected subsets of CpGs based on the statistical significance of their correlation with age as “foreground”. These subsets are not meant to correspond exactly to any of the particular regression models, but to capture the general association of age-related CpGs (from which the regression models are drawn) and biological function inferred via proximity of the CpGs to known genes.

We also partitioned the CpGs into groups with positive (gain of methylation) or negative (loss of methylation) with age, as these two groups have been noted to correspond to separate classes of biomolecular function in previous work [17, 59]. As negatively correlated sites generally partition to distal parts of gene bodies or inter-genic regions, they tend to have limited annotation. Conversely, positively correlated sites localize to promoter regions of genes for which there is generally more detailed annotation. To ensure the selection of statistically significant age-related CpGs, we performed a multiple-testing correction [60] on the p-values and selected only those with adjusted values <= 0.05. The annotation tool (GREAT) accesses a large and diverse number of databases and function ontologies. Here, we report those results edited down to non-redundant highlights. We found that a subset of 91 negatively-correlated CpGs (0.1% of total) localized to 125 genes that function in cellular organization and the Notch pathway, an evolutionarily conserved cell-to-cell signaling pathway important for cell proliferation and differentiation (Table 1A). The subset of 90 positively-correlated CpGs (0.1% of total) localized to 71 genes with vital roles in embryonic organismal development and chromatin states (Table 1B). In summary, the canid genes whose DNA-methylation changes are most strongly correlated to age (both negatively and positively) are critical developmental genes; those that determine cell fate and organ development in the embryonic stage of life, as has been noted in previous work with DNA-methylation in humans [17, 59].

**Table 1:** Functional enrichment studies of age related CpGs in canids

Functional Annotation	Hypergeometric FDR Q-Value
**A. Functional roles of CpGs that lose methylation with age**	
compartment pattern specification	2.8x10^-4^
proximal tubule development	4.3x10^-4^
carbohydrate derivative transport	8.8x10^-4^
Notch signaling pathway	1.3x10^-3^
**B. Functional roles of CpGs that gain methylation with age**	
regulation of transcription, DNA-dependent	1.6x10^-11^
regulation of RNA biosynthetic process	8.8x10^-12^
organ development	1.0x10^-11^
embryonic organ morphogenesis	1.8x10^-10^
anatomical structure development	8.3x10^-10^
Set 'Suz12 targets': genes identified by ChIP on chip as targets of the Polycomb protein SUZ12 in human embryonic stem cells.	2.6x10^-10^
Genes with high-CpG-density promoters (HCP) bearing the H3K27 tri-methylation (H3K27me3) mark in brain.	6.9x10^-9^
Genes with high-CpG-density promoters (HCP) bearing histone H3 trimethylation mark at K27 (H3K27me3) in neural progenitor cells (NPC).	1.5x10^-8^

## DISCUSSION

More broadly, our study demonstrates that DNA-methylation correlates with age in dogs and wolves as it does in human and related species. This age-dependence of DNA-methylation is conserved at syntenic sites in the respective genomes of these canid species as well for more distantly related mammalian genomes such as human. Strikingly, the age associations of syntenic CpGs is well conserved (r=0.20) even though the data were generated on different platforms (RRBS vs Illumina methylation array). Overall, our study demonstrates that dogs age in a similar fashion to humans when it comes to DNA methylation changes.

Race/ethnicity and sex have a significant effect on the epigenetic age of blood in humans [61]. Further, genetic loci have been found that affect epigenetic aging rates in humans [62]. It will be interesting to determine whether sex effects can also be observed in dogs and whether genetic background relates to the ticking rate of the canid clock. Based on our preliminary blood samples of 108 canid specimens, including both dogs and wolves, we accurately measured the methylation status of several hundred thousand CpGs. We demonstrate that these data can produce highly accurate age estimation methods (epigenetic clocks) for dogs and wolves separately. By first removing sites that were variable between dogs and wolves, we could also establish a highly accurate epigenetic clock for all canids (i.e. dogs and wolves combined). This clock allows us to estimate the age of half the canids to within a year.

Our study has several limitations including the following. First, the sample size was relatively low (n=108). There is no doubt that more accurate clocks could be build based on larger sample sizes. Second, we only focused on blood tissue. Future studies could explore other sources of DNA such as buccal swabs. Third, the chronological ages of the wolves are probably not very accurate since they were estimated by the investigators.

In human studies, we have found that lifestyle factors (e.g. diet) have at best a weak effect on cell-intrinsic epigenetic aging rates measured by the 353 CpG based clock [63]. By contrast, extrinsic measures of epigenetic age acceleration, which also capture age related changes in blood cell composition, relate to lifestyle factors that are known to be protective in humans (e.g. consumption of fish, vegetables, moderate alcohol, and to higher levels of education). Biomarkers of metabolic syndrome were associated with increased DNAm age but we could not detect a protective effect of metformin in this observatio-nal study [63]. The presented canid aging clocks open up the possibility of assessing dietary and pharmacological intervention on canid aging. The genome coordinates for the CpGs and corresponding regression coefficients of our final canid age estimator and of our dog age estimator can be found in Table 2 and Table 3, respectively.

**Table 2:** Multivariate model of canid age

Canine coordinate (canFam3)	Coef	Mean meth	Corr(age,meth)	Human coordinate (hg19)	Proximal genes
Intercept term	4.382				
chr1: 815007	−0.405	0.95	−0.27	chr18: 77637184	KCNG2 (+13517), PQLC1 (+74479)
chr1: 48720985	0.5191	0.95	0.31		
chr1: 49472858	0.2594	0.64	0.28		
chr1: 90590933	−0.0041	0.94	−0.32	chr9: 1872401	Intergenic
chr1: 98573761	−0.0837	0.6	−0.42		
chr1: 98573781	−0.1991	0.2	−0.37		
chr1: 101051499	−0.2786	0.79	−0.4	chr19: 57398441	ZIM2 (−46345)
chr1: 108136920	0.933	0.98	0.22	chr19: 48626542	PLA2G4C (−12469), LIG1 (+47317)
chr1: 117122008	−0.2091	0.82	−0.33	chr19: 36035408	TMEM147 (−1088)
chr1: 117495962	−0.1664	0.86	−0.41	chr19: 35540744	FXYD3 (−66421), HPN (+9335)
chr1: 121791246	−0.3188	0.67	−0.38	chr19: 30153492	PLEKHF1 (−2470)
chr1: 121796139	0.3134	0.96	0.28		
chr1: 121864927	0.3367	0.83	0.3	chr19: 30042558	POP4 (−52365), VSTM2B (+25153)
chr2: 10101121	−0.0266	0.65	−0.29		
chr2: 30853505	−0.0048	0.93	−0.28	chr10: 4714389	Intergenic
chr2: 36347652	0.0248	0.37	0.36	chr5: 140749805	PCDHGA6 (−3845), PCDHGB3 (−25)
chr2: 71080824	0.0288	0.86	0.33	chr1: 30051475	Intergenic
chr2: 82210243	−0.387	0.94	−0.22	chr1: 15602565	Intergenic
chr2: 84377388	−0.4829	0.97	−0.36	chr1: 11951757	NPPB (−32770), KIAA2013 (+34722)
chr2: 84445018	0.0924	0.26	0.33	chr1: 11864680	CLCN6 (−1587), MTHFR (−1379)
chr3: 1128258	−0.0993	0.94	−0.37		
chr3: 51442070	−0.0641	0.8	−0.3	chr15: 88733456	NTRK3^d^ (+66204)
chr3: 60468935	−0.2449	0.82	−0.43	chr4: 8834358	HMX1 (+39184)
chr3: 62880832	0.4931	0.88	0.25	chr4: 17638199	MED28 (+21946)
chr3: 84450199	−0.0696	0.89	−0.49	chr4: 25978965	SMIM20 (+63140)
chr4: 28034141	−0.6356	0.14	−0.33	chr10: 79971431	Intergenic
chr4: 28162022	−0.1129	0.89	−0.32	chr10: 80116134	Intergenic
chr4: 28489863	0.0289	0.7	0.24	chr10: 80479452	Intergenic
chr4: 79153238	0.1058	0.09	0.42	chr5: 27038840	CDH9 (−148)
chr5: 4750111	−0.089	0.35	−0.5	chr11: 129969307	ST14 (−60149), APLP2 (+29507)
chr14: 41413362	−0.2829	0.57	−0.33	chr7: 28355716	CREB5 (−96427)
chr14: 59995975	−0.0142	0.73	−0.34	chr7: 121776852	AASS (−2977)
chr15: 17780647	1.3988	0.97	0.29	chr14: 20915434	TEP1 (−33855), OSGEP (+7829)
chr15: 17785631	−0.3897	0.94	−0.43	chr14: 20921454	APEX1 (−1899), OSGEP (+1809)
chr16: 131577	−0.0629	0.73	−0.31		
chr16: 247019	0.8011	0.83	0.34		
chr17: 18033866	0.2802	0.82	0.32	chr2: 23704553	Intergenic
chr18: 1791242	−0.0118	0.94	−0.35	chr7: 50515762	FIGNL1 (+1659)
chr18: 25850449	−0.2607	0.92	−0.34		
chr18: 33813035	−0.1549	0.78	−0.41	chr11: 33962891	LMO2 (−49056)
chr18: 43740411	−0.1646	0.94	−0.34	chr11: 45669463	CHST1 (+17708)
chr18: 48905778	0.0765	0.72	0.41	chr11: 68925723	Intergenic
chr18: 49633631	−0.0336	0.49	−0.22	chr11: 67984189	SUV420H1 (−3308)
chr18: 53920336	−0.0957	0.8	−0.4		
chr20: 44455198	0.3338	0.72	0.46		
chr20: 49366316	−0.0004	0.71	−0.28	chr19: 12895268	HOOK2 (−8932), JUNB (−7041)
chr21: 23088752	−0.4109	0.88	−0.28	chr11: 75219103	GDPD5 (+17844)
chr21: 47917499	−0.0235	0.84	−0.29	chr11: 27349807	Intergenic
chr22: 56299850	−0.2509	0.89	−0.32	chr13: 108022629	Intergenic
chr23: 24782165	−0.1049	0.89	−0.33	chr3: 18277027	Intergenic
chr23: 24782179	−0.658	0.94	−0.31	chr3: 18277013	Intergenic
chr24: 42551119	−0.2892	0.93	−0.35	chr20: 56148739	PCK1 (+12604), ZBP1 (+46789)
chr24: 45589901	0.2135	0.97	0.27	chr20: 59877087	CDH4^b^ (+49606)
chr26: 220859	0.0037	0.98	0.27		
chr26: 5991914	−0.2154	0.93	−0.37	chr12: 124138408	TCTN2 (−17251), GTF2H3 (+20033)
chr26: 11457252	−0.3679	0.94	−0.28	chr12: 114784708	TBX5^a^^,^^b^^,^^c^^,^^d^ (+61538)
chr26: 37645878	−0.4125	0.93	−0.3		
chr27: 1189935	−0.0497	0.78	−0.39	chr12: 54471815	HOXC4^a^^,^^b^^,^^c^^,^^d^ (+24155)
chr27: 2886690	−0.0004	0.86	−0.44	chr12: 52559286	KRT80 (+26497), C12orf44 (+95532)
chr27: 45394279	−0.4689	0.93	−0.45		
chr28: 23823079	−0.2956	0.91	−0.31		
chr28: 40564054	−0.0603	0.96	−0.33	chr10: 134593678	NKX6-2^a^^,^^b^^,^^c^^,^^d^ (+5877)
chr30: 15275091	−0.0557	0.89	−0.31		
chr30: 27934524	−0.0484	0.93	−0.26	chr15: 63648005	CA12 (+26354), APH1B (+78253)
chr30: 38620897	0.8567	0.94	0.33	chr15: 78043186	Intergenic
chr31: 27720671	0.0332	0.16	0.46	chr21: 34444104	OLIG1^a^^,^^d^ (+1655)
chr31: 36955453	−0.5267	0.91	−0.36	chr21: 44079991	PDE9A (+6127)
chr31: 37492782	−0.4942	0.49	−0.56		
chr32: 1431916	−0.2835	0.07	−0.3	chr4: 77752402	Intergenic
chr32: 38110814	−0.076	0.87	−0.35		
chr33: 22992599	−0.0003	0.93	−0.34	chr3: 119042586	ARHGAP31 (+29367)
chr33: 25783582	0.3819	0.98	0.34	chr3: 122422615	PARP14 (+23151), HSPBAP1 (+90055)
chr33: 31142995	−0.8311	0.95	−0.3	chr3: 194291430	ATP13A3 (−72338), TMEM44 (+62719)
chr34: 40858941	−0.1582	0.93	−0.38	chr3: 177096996	Intergenic
chr35: 2307155	−0.1171	0.94	−0.32	chr6: 1886203	Intergenic
chr36: 2545193	0.2403	0.84	0.47	chr2: 157179898	NR4A2 (+9329)
chr36: 19969591	0.0515	0.27	0.38	chr2: 177025691	HOXD1^a^^,^^b^^,^^c^^,^^d^ (−27615), HOXD4^a^^,^^b^^,^^c^^,^^d^ (+9742)
chr37: 6301	−0.1058	0.69	−0.43		
chr37: 25454687	0.4555	0.32	0.33	chr2: 219736500	WNT10A^a^^,^^b^^,^^c^^,^^d^ (−8584), WNT6^a^^,^^b^^,^^c^^,^^d^ (+11957)
chr38: 16230281	0.1055	0.92	0.23	chr1: 221912099	DUSP10 (+3418)
chr38: 22365525	−0.1451	0.89	−0.36	chr1: 159724037	CRP (−39659), DUSP23 (−26755)
chr38: 22792877	0.7599	0.72	0.43	chr1: 159145579	DARC (−29621), CADM3^d^ (+4181)
chrX: 80013740	−0.133	0.85	−0.29		

Genome coordinates and coefficient values for predicting a log (base e) transformed version of chronological age. These coefficients were found by regressing a log-transformed version of age on the RRBS DNA-methylation measured from 108 canid blood samples. Since chronological age was log-transformed prior to regression, it is important to exponentiate the age estimate from this model to arrive at age estimates in units of years. We provide the mean methylation and Pearson correlation with age for each individual CpG. Where possible, we identify, via synteny to the human genome, genes that are proximal to the CpGs in our models. Numbers in parentheses are the distance in bases to the Transcription Start Site of the gene. Additionally, we note those genes with experimentally inferred relevance to cellular identity (pluripotency).

Genes experimentally identified as targets of pluripotency factors and the Polycomb repressor complex [69, 70]

a genes identified by ChIP on chip as targets of the Polycomb protein EED in human embryonic stem cells.

b genes possessing the trimethylated H3K27 (H3K27me3) mark in their promoters in human embryonic stem cells, as identified by ChIP on chip

c Polycomb Repression Complex 2 (PRC) targets; identified by ChIP on chip on human embryonic stem cells as genes that: possess the trimethylated H3K27 mark in their promoters and are bound by SUZ12 and EED Polycomb proteins

d genes identified by ChIP on chip as targets of the Polycomb protein SUZ12 in human embryonic stem cells

**Table 3:** Multivariate model of domesticated dog age

Canine coordinate (canFam3)		Coef	Mean Meth	Corr(age,meth)	Human coordinate (hg19)	proximal genes
(Intercept)	−6.9009					
chr1: 98804509	−0.1771	0.92	−0.56	chr9: 95371248	ECM2 (−72912), IPPK (+61298)	
chr2: 34467253	−0.1762	0.96	−0.52	chr10: 323319	Intergenic	
chr2: 50165769	1.8403	0.97	0.56	chr5: 63460330	RNF180 (−1378)	
chr3: 54128482	0.1444	0.77	0.51	chr15: 85429683	PDE8A^a^^,^^b^ (−93987), SLC28A1 (+1771)	
chr4: 28320267	−0.4038	0.94	−0.43	chr10: 80292869	Intergenic	
chr5: 19204758	−0.5393	0.31	−0.62	chr11: 114000061	ZBTB16^a^^,^^b^^,^^c^^,^^d^ (+69747)	
chr5: 32946701	0.1414	0.63	0.67	chr17: 8027247	ALOXE3^b^^,^^d^ (−4883), HES7^a^^,^^b^^,^^c^^,^^d^ (+154)	
chr5: 57889544	0.1104	0.88	0.45	chr1: 3202081	Intergenic	
chr6: 31347568	−0.0245	0.64	−0.5	chr16: 11536754	ENSG00000188897 (+80689), RMI2 (+97467)	
chr6: 77030251	0.5807	0.82	0.6	chr1: 68732333	WLS^a^^,^^d^ (−34106)	
chr7: 54060517	0.3047	0.06	0.48	chr18: 33708261	ELP2 (−1599), SLC39A6 (+1019)	
chr8: 50434546	0.0351	0.91	0.42	chr14: 78126349	SPTLC2 (−43234), ALKBH1 (+48013)	
chr9: 58841067	0.318	0.15	0.61	chr9: 126779366	LHX2^a^^,^^b^^,^^c^^,^^d^ (+5478)	
chr10: 21355951	0.3876	0.86	0.52			
chr10: 55453590	−0.4182	0.95	−0.75	chr2: 54776879	SPTBN1 (+93458)	
chr10: 56694481	0.4854	0.09	0.46	chr2: 56151248	EFEMP1 (+25)	
chr10: 62832468	0.2077	0.77	0.6	chr2: 63279783	OTX1^a^^,^^b^^,^^c^^,^^d^ (+1847)	
chr10: 62832512	0.2347	0.58	0.67	chr2: 63279827	OTX1^a^^,^^b^^,^^c^^,^^d^ (+1891)	
chr11: 4422863	0.7296	0.91	0.44	chr5: 113831979	Intergenic	
chr11: 56812470	0.0579	0.87	0.47	chr9: 102590004	NR4A3^a^^,^^b^^,^^c^^,^^d^ (+996)	
chr11: 68812130	1.204	0.95	0.5	chr9: 117441733	C9orf91 (+68248)	
chr12: 67228192	1.4284	0.98	0.39	chr6: 110931985	Intergenic	
chr12: 67482078	−0.2241	0.19	−0.53	chr6: 111267687	GTF3C6 (−12075), AMD1 (+71715)	
chr12: 67482081	−0.0314	0.26	−0.48	chr6: 111267690	GTF3C6 (−12072), AMD1 (+71718)	
chr12: 71716109	0.0065	0.82	0.34			
chr14: 8324605	0.3087	0.65	0.59	chr7: 127670876	LRRC4 (+246)	
chr14: 34373167	0.2091	0.76	0.64	chr7: 20371740	ITGB8 (+995)	
chr18: 36757482	−0.403	0.83	−0.63	chr11: 30565405	MPPED2 (+36637)	
chr19: 22964392	−0.0157	0.89	−0.54	chr2: 128408298	GPR17 (+4860), LIMS2 (+13821)	
chr20: 43010636	0.4045	0.97	0.5			
chr20: 44455198	0.0664	0.72	0.47			
chr20: 56821233	1.2199	0.98	0.42	chr19: 2210771	SF3A2^a^ (−25748), DOT1L (+46624)	
chr22: 49950553	0.1423	0.54	0.63	chr13: 100636088	ZIC2^a^ (+2063)	
chr24: 37978456	−0.0979	0.77	−0.59			
chr28: 39644410	0.0856	0.84	0.37			
chr30: 38620897	1.4441	0.94	0.51	chr15: 78043186	Intergenic	
chr31: 37492782	−0.0028	0.47	−0.51			
chr33: 23073877	−0.0296	0.96	−0.52	chr3: 119134135	TMEM39A (+48393)	
chr34: 40999085	−0.1395	0.97	−0.46	chr3: 177284378	Intergenic	
chr36: 2545142	0.432	0.5	0.62	chr2: 157179848	NR4A2 (+9379)	

Genome coordinates and coefficient values for predicting a log (base e) transformed version of chronological age. These coefficients were found by regressing a log-transformed version of age on the RRBS DNA-methylation measured from 46 domesticated dog blood samples. Since chronological age was log-transformed prior to regression, it is important to exponentiate the age estimate from this model to arrive at age estimates in units of years. We provide the mean methylation and Pearson correlation with age for each individual CpG. Where possible we identify, via synteny to the human genome, genes that are proximal to the CpGs in our models. Numbers in parentheses are the distance in bases to the Transcription Start Site of the gene. Additionally, we note those genes with experimentally inferred relevance to cellular identity (pluripotency).

Genes experimentally identified as targets of pluripotency factors and the Polycomb repressor complex [69, 70]

a genes identified by ChIP on chip as targets of the Polycomb protein EED in human embryonic stem cells.

b genes possessing the trimethylated H3K27 (H3K27me3) mark in their promoters in human embryonic stem cells, as identified by ChIP on chip

c Polycomb Repression Complex 2 (PRC) targets; identified by ChIP on chip on human embryonic stem cells as genes that: possess the trimethylated H3K27 mark in their promoters and are bound by SUZ12 and EED Polycomb proteins

d genes identified by ChIP on chip as targets of the Polycomb protein SUZ12 in human embryonic stem cells

## METHODS

### Reduced representation bisulfite sequencing (RRBS)

We obtained previously published canine RRBS methylation data as CGmap files (see Janowitz, Koch, et al. 2016) [50]. Both wolf and dog data were aligned to the canine genome (canFam3).

### Data processing

For each CpG site in each sample we estimated the methylation frequency as the number of methylated mapped read counts over the total mapped read counts and computed a corresponding 95% confidence interval from the binomial distribution [64]. For inclusion in our analysis, we required that each CpG site had confident methylation frequencies in at least 95% of samples. Confidence was defined as having a confidence interval smaller than 0.63 (roughly equivalent to requiring a minimum of 15 mapped reads at that site). For the remaining elements in the data matrix, we used the frequencies calculated regardless of confidence or imputed missing values using R package “softImpute” with type option “ALS” [65].

### Culling species-specific differential methylation

To exclude species-specific differential methylation as a confounder, we first constructed a methylation matrix with no dog samples with ages greater than the maximum observed wolf age (8 years). For each CpG we then computed a t-test of the dog methylation values versus the wolf methylation values and excluded those with t >= 2 from use in regression modelling.

### Computing age correlations for DNA methylation

When comparing age-correlations computed in datasets of different sizes, we use a z-score instead of the Pearson correlation coefficient. A Student t-test statistic for testing whether a Pearson correlation (*r*_s_) is different from zero is given by

$$Z_{s}\;=\;\frac{\sqrt{m_{s}\;-\;2}\cdot r_{s}}{\sqrt{1\;-\;r_{s}{}^{2}}}$$

where m_s_ denotes the number of observations (i.e. samples) in the s-th data set.

### Regression

Penalized regression models were built using glmnet [66]. Given that we would like to see a reduction in the number of predictors from potentially hundreds of thousands of CpGs as input, we utilized the “elastic net” version of glmnet corresponding to an alpha parameter of 0.5. For all results reported here, the internally cross-validated (cv.glmnet) was utilized to automatically select the optimal penalty parameter.

### Functional annotation and multi-species synteny

Canid methylation sites (using coordinates from the CanFam3 draft genome) were first mapped to the human genome (hg19) where possible so that functional analysis tools with access to the most complete and detailed annotations could be utilized. This mapping was made using the "liftOver" tool and associated human to canine chain files available at the UCSC Genome Browser[67]. The human genome coordinates were then used as input to the Genomic Regions Enrichment of Annotations Tool (GREAT) [68].
